# Designing a Placebo Microneedle Stamp: Modeling and Validation in a Clinical Control Trial

**DOI:** 10.3390/pharmaceutics16030395

**Published:** 2024-03-14

**Authors:** Seung-Yeon Jeong, Ye-Seul Lee, Ji-Yeun Park, Jung-Hwan Park, Hi-Joon Park, Song-Yi Kim

**Affiliations:** 1 Department of Anatomy and Acupoint, College of Korean Medicine, Gachon University, Seongnam 13120, Republic of Korea; seungyeonj1993@gmail.com; 2 Jaseng Spine and Joint Research Institute, Jaseng Medical Foundation, Seoul 06110, Republic of Korea; 3 Department of Meridian & Acupoint, College of Korean Medicine, Daejeon University, Daejeon 34141, Republic of Korea; 4 Department of BioNano Technology, Gachon BioNano Research Institute, Gachon University, Seongnam 13120, Republic of Korea; 5 Acupuncture and Meridian Science Research Center (AMSRC), Kyung Hee University, Dongdaemun-gu, Seoul 02447, Republic of Korea

**Keywords:** microneedles, placebo, sham control, drug delivery, acupuncture

## Abstract

Recently, several clinical studies have been conducted using microneedles (MNs), and various devices have been developed. This study aimed to propose and confirm the feasibility of a placebo control for activating MN clinical research. A 0.5 mm MN stamp with 42 needles was used as a treatment intervention, and a placebo stamp with four acupressure-type needles that did not penetrate was proposed and designed as a control for comparison. First, to check whether the placebo stamp did not invade the skin and to set an appropriate level of pressure to be provided during skin stimulation, two participants were stimulated with five different forces on the forearm, and then the skin was dyed. Secondly, to evaluate the validity of the placebo control group, a blinded study between the MN and placebo stamps was performed on 15 participants. We confirmed that the placebo stamp did not penetrate the skin at any intensity or location. Both types of stamps reported relatively low pain levels, but the MN stamp induced higher pain compared to the placebo stamp. Based on the speculation regarding the type of intervention received, the MN stamp was successfully blinded (random guess), whereas the placebo stamp was unblinded. However, according to a subgroup analysis, it was confirmed that the group with low skin sensitivity was completely blind. Blinding the placebo MN stamp had limited success in participants with low skin sensitivity. Future research on suitable placebo controls, considering the variations in MN stamp length and needle count, is warranted.

## 1. Introduction

Microneedles (MNs) are systems that intradermally deliver drug components using single or an array of micrometer-sized needles that penetrate the epidermis or epithelial layers of the skin [1]. MNs are not only used for medical purposes, such as disease diagnosis and treatment, but also in the beauty industry. Recently, research and development on MNs have increased [2,3].

**Figure 1:**
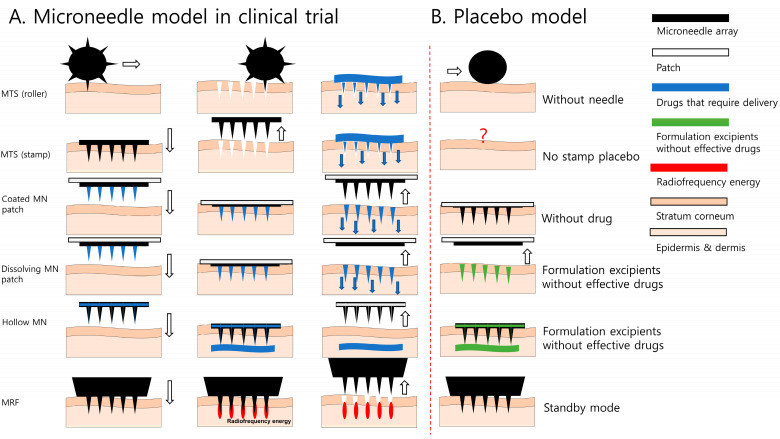
Microneedle models used for cosmetic and therapeutic purposes. (**A**) Microneedle models in clinical trial; (**B**) placebo model in microneedle studies. MN, microneedle; MRF, microneedle radiofrequency; MTS, microneedle therapy system. The white arrow indicates the direction the microneedle is moving.

Depending on the type of device, MNs can be classified as MN therapy systems (MTSs), MN array patches (MAPs), MN radiofrequency (MRF), hollow MN, and MN monitors, and the application method is different for each type [4]. Various types of MNs can be considered similar in terms of their utilization of micron-sized needles; however, their roles may differ slightly. For example, although MNs are an efficient route for subcutaneous drug delivery [5], they are sometimes used to induce spontaneous skin regeneration by providing physical stimulation to the skin using the MN itself, without drugs [6]. Among the many forms of MTS, MN rollers and stamps are used in both of these roles; they are mainly used in pretreatment for the topical application of drugs or cosmetics, or for skin stimulation without drugs to generate collagen [7]. Another type of MN, the MAP, specializes in vaccine delivery in the form of a patch of drug-containing or drug-coated MN arrays [8]. MRF transmits heat energy through radiofrequency after skin stimulation and is mainly used for skincare [9]. Hollow MNs are a form of drug delivery through microchannels and are mainly used for drug delivery, such as insulin or vaccines; they employ rapid drug absorption and continuous drug concentration control technologies [10] (Figure 1A).

For these various types of MNs to be used medically, a high level of evidence is required to prove their effectiveness, safety, and cost-effectiveness. In particular, results obtained through experimental studies, such as randomized controlled trials (RCTs), are considered important because they strongly suggest that observed changes after treatment are caused by intervention (commonly expressed as having high internal validity) [11]. Previous RCTs evaluating the effectiveness of MNs have shown that usual treatments, considered the gold standard for disease, have been primarily applied as control groups, and in rare cases, as placebo controls. For example, laser therapy [12] and cryotherapy [13] have been used as controls for MNs to treat local skin diseases and improve wrinkles. To observe whether pretreatment with MNs is effective for drug delivery, only drugs without pretreatment with MNs were used as controls [14]. In drug delivery studies involving systemic effects, such as insulin or vaccine delivery, intramuscular [15], intradermal [16], and subcutaneous [17] injections were mainly used as controls. In this way, when comparing whether the “pretreatment process” of MN is key to effective drug delivery, a no-treatment group (drug delivery without MN pretreatment) was used as a control instead of an active control. However, placebo or sham controls have rarely been used in clinical studies that use MNs as treatment interventions.

Placebo controls are useful for assessing the specific efficacy of MNs, excluding placebo effects known to be caused by expectations for treatment or doctor–patient relationships in the course of treatment [18]. In general, a placebo control is defined as one that should be similar in appearance to make individuals feel that they are actually receiving treatment, but it must not cause any physiological effects [18,19]. The types of placebo controls for MNs used in previous studies and the limitations of each placebo control are as follows: first, the same MNs were used in both comparison groups; however, only the drug in the placebo control group was replaced with an inactive drug [8]. In this case, only the placebo effect of the drug and not the placebo effect of the MNs can be eliminated. Second, in MNs such as MRF, “stand-by mode”, which limits additional stimulation (i.e., electrical and radiofrequency) applied to MNs, was used as a placebo control [20]. This has the potential to cause an apparent difference in the sensation provided by the treatment and placebo control groups; therefore, there can be a high risk of performance bias caused by guessing which treatment the individual received on his/her own. Third, to minimize the physical stimulation of MNs, a placebo control, in which the needles were removed, was used in the study of MN rollers. However, even in this case, the blinding of patients was never evaluated; therefore, it is difficult to rule out the possibility of performance bias caused by the participants’ knowledge of the treatment assigned to them during the study period [21]. Additionally, there have been no studies in which a placebo control was used for MTS in a stamp form (Figure 1B).

Therefore, the purpose of this study was to (1) propose a placebo model for the MN stamp that can be used in clinical studies to clearly evaluate the efficacy of MN and confirm its validity; and (2) determine whether the placebo control was indistinguishable from the MN stamp in normal human individuals through a blinding assessment.

## 2. Materials and Methods

This study was conducted as follows: first, we created a placebo stamp that is noninvasive and provides only minimal stimulation but cannot be distinguished from the MN stamp device. Next, we confirmed that the placebo stamp did not penetrate the skin, and the experimental conditions for the blind test were set. Finally, a blind test for the recruited participants was performed to compare the sensory type and intensity of stimulation between the commercial MN and placebo stamps.

### 2.1. Modeling Placebo Stamp

#### 2.1.1. Placebo Stamp Concept

We set the following conditions to fabricate a placebo stamp that can be applied as a control for the MN stamp, consisting of 42 sterilized surgical stainless-steel needles with a length of 0.5 mm (Dermaroller System, 40p derma stamp, Gwangju-si, Republic of Korea), which is commercialized for skincare purposes. There should be no physiological effect after stimulation of the placebo stamp, and the skin sensation felt by the participant (e.g., skin contact area of the stamp, pain due to needle stimulation, stimulation intensity, and time) and appearance perceived by practitioners and the participants should be similar between the MN and placebo stamps.

Under these conditions, the placebo stamp model was designed to have the same size as the skin contact area of the MN stamp, and an outer cover was created to make the MN stamp indistinguishable from the placebo stamp model. They were designed and manufactured using three-dimensional printing technology. Several designs have been proposed and experimentally confirmed to reduce the difference between pain and stimulation sensations.

#### 2.1.2. Configuration of Placebo MN Stamp Design

In order for the placebo stamp to induce a sensation similar to the MN stamp while minimizing the actual amount of stimulation, we considered two types of skin stimulation: “method of inducing pain sensation similar to physical contact by instantaneously delivering low temperature to the skin” and “method to induce pain sensation similar to a MN stamp by applying a maximum of noninvasive physical contact”.

To confirm the feasibility of these two methods, we first implemented a model that induced pain at a low temperature. The experimental implementation and evaluation were conducted by varying the materials in contact with the skin, such as plastic and metal, the size of the area in which a low-temperature object touched the skin, and the temperature of the object in contact with the skin. As a result, this type of placebo model was excluded from our options because it requires many resources to keep the temperature constant and low enough to induce a sensation similar to the pain caused by the MN stamp, and it is difficult to control the practitioner’s performance bias. 

**Figure 2:**
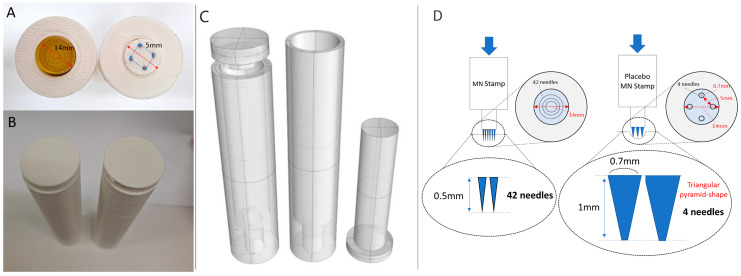
Placebo stamp design. (**A**) Two-intervention bottom design, MN stamp on the left (42 needles, 0.5 mm) and placebo stamp on the right (4 needles, 1.0 mm); (**B**) designed to be indistinguishable for researchers and participants; (**C**) 3D-printed drawing of the microneedles’ outer cover. On the left is the outer cover and lid of the MN stamp, and on the right is the cover of the placebo stamp; (**D**) the difference between the MN stamp and placebo stamp that occurred during the manufacturing process. MNs, microneedles. The blue arrow indicates the direction of force of the microneedle stamp towards the skin.

In the second method, a placebo stamp was designed in the form of an acupressure stamp by modifying noninvasive sham acupuncture. For the material, we chose stainless-steel, a metal that is durable enough to withstand pressure and is similar to MN stamps. Additionally, a triangular pyramid-shaped needle, rather than a hemispherical, cylindrical, or oval needle, was used to provide a feeling similar to that of the MN stamp needle at the moment of touch. After several attempts, considering the problem of 3D printing technology and the sense of the contact surface, a placebo MN stamp in the form of four needles with a diameter of 0.7 mm and length of 1 mm arranged at intervals of 5 mm was designed (Figure 2 and Supplementary Table S1).

#### 2.1.3. Confirmation of Skin Penetration of Placebo MN Stamp and Setting of Experimental Conditions for Blind Testing

We conducted a pilot test to confirm the noninvasiveness of the designed placebo stamp and set the stimulus intensity to minimize the difference in the sensation of stimulation between the two types of stamps. For this purpose, two healthy participants (one male and one female) >19 years old, who had no tactile abnormalities and no resistance to acupuncture, were recruited (we checked for a history of breast cancer to minimize the potential risk of methylene blue dye (MBD), but this risk is mainly caused by injection; therefore, it was not an essential process in this study) [22]. The participants were seated comfortably in a chair, the palms of the armrests were raised upward, and five positions were set at equal intervals between the cubital and wrist creases (Supplementary Figure S1). Afterwards, the MN and placebo stamps were stimulated with a total of five intensities (0.3 kgf, 0.4 kgf, 0.6 kgf, 0.8 kgf, and 1.2 kgf) on both arms. The stimulation site, stimulation intensity, and type of MN stamp were randomly assigned by an independent researcher using a random number table in Microsoft Excel 2016 (Microsoft Corp., Redmond, WA, USA). The stimulation intensity was quantified by an experienced researcher applying a set level of force from the top of the MN stamp using an algometer (push–pull force gauge, BASELINE^®^, Fort Collins, CO, USA).

Immediately after the skin stimulation, the stimulation site was marked, and 200 μL of 1% *w*/*v* MBD was applied to the marked area for 5 min, and then washed with cold running water [23]. Each stained area was photographed using a camera (EOS 7D Mark2, Canon, Ayase, Japan) immediately after the stimulation (0 h) and at 2, 4.5, and 7 h after stimulation. To check for the presence and intensity of needle invasion in the photographed images, two researchers individually counted the number of stained spots caused by needle invasion and analyzed the average value.

### 2.2. Validation Test for Placebo MN Stamp

**Figure 3:**
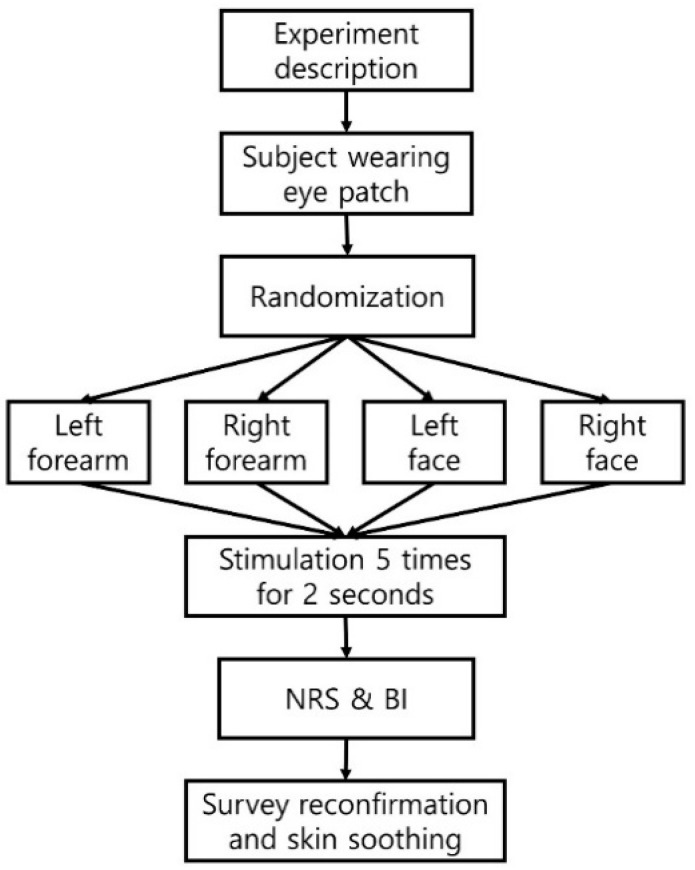
Process of blinding assessment. BI, blinding index; NRS, numeral rating scale.

A double-blind randomized controlled trial was performed to evaluate the validity of the placebo MN stamp (Figure 3). This pilot study was approved by the Gachon University Institutional Review Board (1044396-202008-HR-156-01) and was conducted according to the approved protocol. The criteria for the selection of participants were healthy adults aged >19 years old with no tactile abnormalities or resistance to acupuncture. Participants who had a history of skin reaction-related diseases or needle phobia or were left-handed were excluded from the study. The sample for this study was recruited through public advertisements within the university where the study was conducted, and the number of participants was not to exceed 15 [24,25]. All the participants voluntarily provided written informed consent after being informed of the purpose and method of the study.

**Figure 4:**
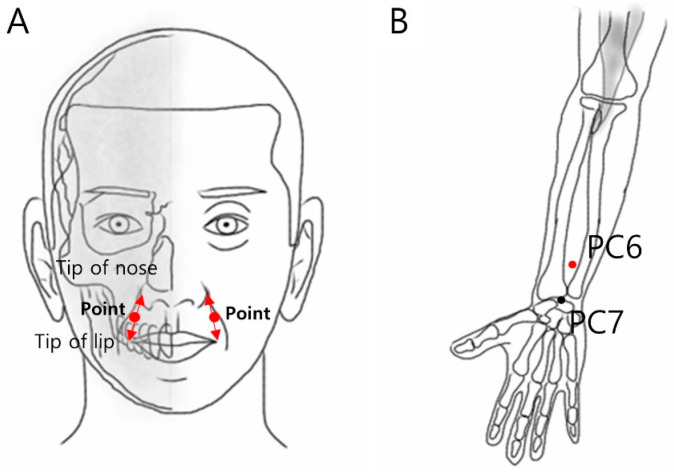
The location of the skin stimulation. (**A**) The location of the facial stimulation, the middle of the tip of the lips and the tip of the nose; (**B**) the location of the forearm stimulation, PC6.

Two areas were selected for stimulation: the face (the central part between the edge of the lips and the edge of the nose) and forearm (acupuncture point PC6; front side of the forearm), which are clinically used for MN stamps or acupuncture and are also sensitive areas (Figure 4). In all the procedures, the participants covered their eyes using a sleep mask to blind them to the device. For forearm stimulation, the entire forearm was placed on a desk with the palm facing up, and the participant’s head was fixed to the wall to maintain a stable state. The sequences of stimulation sites (left or right forearm and face) were randomly assigned using a computer random number table managed by an independent researcher. A practitioner who received the random number table stimulated the skin five times for 2 s with one of the MN stamps of the same shape, marked as “No. 1” or “No. 2” on the patient with a pressing force (0.6 kgf) of the preset strength in the pilot study, while maintaining concealment of the allocation. To maintain blinding, the practitioner was trained not to check the bottom of the MN stamps until the end of the experiment. After the stimulation was completed, the irritated skin was soothed with an ice roller. Additionally, the participants responded to a self-report questionnaire with a band-aid attached to the stimulation area. The following information was collected from the participants.

#### 2.2.1. Baseline Characteristics and Skin Sensitivity by Two-Point Discrimination (TPD)

Demographic information on the age, sex, height, weight, drinking, and smoking status of the included participants was investigated. Before the blinding assessment began, the TPD threshold was measured to check the skin sensitivity of the participants. A participant sat in a comfortable chair with both palms facing up and wore a sleep mask so that the measurement site was not visible. The TPD threshold was measured using a two-point discriminator (12-1480, Baseline, USA) on the stimulation area of the participant’s forearm and face, and it was assumed that the lower the threshold, the higher the skin sensitivity. For the TPD threshold, both ascending and descending series were used for both sites [26]. For the ascending series, the distance between the two points started at 10 mm (forearm) or 5 mm (face), and the distance between the two points was gradually increased by 2 mm (forearm) or 1 mm (face) until it was indicated that the participant experienced ‘one point’ [27]. In the descending series, the distance between the two points, which began at a separation of 25 mm (forearm) or 15 mm (face), was gradually reduced by 2 mm (forearm) or 1 mm (face) increments until the participant indicated that ‘one point’ was experienced. Finally, the TPD value was defined as the average of the measurements obtained using the two methods [28].

#### 2.2.2. Blinding Assessment of Placebo MN Stamp

After stimulation, the participants evaluated the pain at each stimulation site using a numerical rating scale (NRS) between 0–10, with 0 indicating no pain and 10 indicating the worst pain. Blinding assessment was performed using the blinding index (BI) by having the participant guess one of ‘MN stamp’, ‘Acupressure needle stamp’, or ‘I don’t know’ for the stimulation tool used in each area. Each participant was asked to freely draw the number, shape, and configuration of the stimulation device needles.

#### 2.2.3. Statistical Analysis

All statistical analyses were performed using IBM Statistical Product and Service Solutions (SPSS) Statistics 25 (IBM Industry, Armonk, NY, USA). Nonparametric methods were used because of the small sample size. For the stimulation intensity analysis, Spearman’s correlation was used to analyze the correlation between stimulation intensity, location, and the number of invasion point. Pain analysis was performed using an NRS between 0–10, and Bang’s BI was used to evaluate the success of blinding [29]. Through a demographic survey and TPD analysis, pain and BI were analyzed using the Wilcoxon signed–rank test, according to age (20–30 years old, young age group; >60 years old, old age group) and sensitivity difference (for each TPD value, the highest-scoring seven participants were set as the sensitive group, and the lowest-scoring eight participants were the low-sensitivity group). The sensitivity group was divided based on the average left and right TPD measurements of the forearm and face. The BI was calculated for the MN and placebo stamps separately using 95% confidence intervals (CIs). Blinding status was determined by considering the following: BI ≥ 0.2 unblinded; −0.2 < BI < 0.2 random guesses; and BI ≤ −0.2 opposite guesses [30]. Data were summarized using means and standard deviations, and all *p*-values were considered significant at <0.05.

## 3. Results

### 3.1. Invasion Comparison and Stimulation Intensity Setting

**Figure 5:**
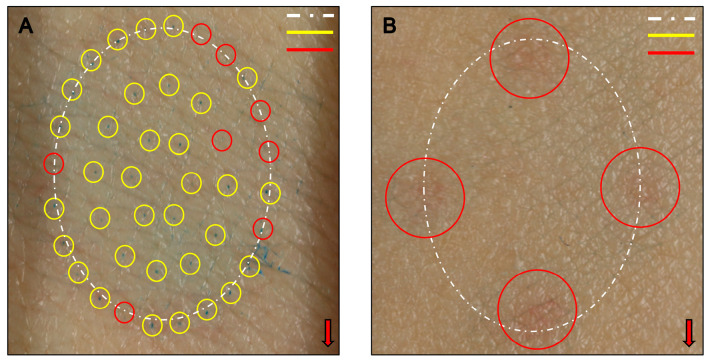
Healthy human skin, in which the invasiveness of the stamp was visually confirmed by methylene blue staining (*n* = 2). (**A**) Microneedle stamp application; (**B**) placebo microneedle stamp application. The arrow’s direction indicates the trunk side. A white dashed–single dotted line denotes the skin contact area outside the MN stamp; yellow circles represent areas stained with MN penetration; red circles represent unstained areas where needles did not penetrate into the skin.

As a result of an experiment on two participants to confirm invasion and set stimulation intensity, needle penetration was confirmed in the case of the MN stamp (Figure 5A and Supplementary Figure S2), whereas the placebo stamp did not show any stained areas indicative of skin penetration across all sites, intensities, and measurement times (Figure 5B). This indicates that the needle of the placebo stamp did not invade the skin. In the case of the MN stamp, there was no correlation between the intensity of stimulation and the degree of invasion (r = −0.588, *p* = 0.074; Supplementary Table S2). When both participants were stimulated with an intensity of 0.3 kgf, the degree of invasion was the least, and both participants showed that the invasion rate increased as the stimulation intensity increased. However, when the stimulation strength increased by >0.6 kgf, the invasion rate decreased (Supplementary Figure S2). Another variable that could affect invasion, the stimulation site, showed no correlation with the degree of invasion (r = −0.302, *p* = 0.397; Supplementary Table S2).

### 3.2. Validation Test

#### 3.2.1. Patients’ Characteristics

Fifteen participants met the inclusion criteria and participated in the study. The participants were randomly assigned to each site and stimulation device and filled out a questionnaire after stimulation. Nine participants were aged 20–30 years old, and six were aged 60–71 years (Table 1). Accordingly, the participants aged 20–30 years were classified into a young age group, and those aged ≥60 years were classified into an old age group. The male ratios in the young and old age groups were 55.6% and 16.7%, respectively, and the male ratio was significantly higher in the young age group (*p* = 0.046). As a result of the TPD of all the participants, the forearm was statistically higher than that of the facial area (38.7 ± 29.2 vs. 10.9 ± 3.7, *p* < 0.05), which was the same in young and old age groups. In the case of forearm TPD, the old age group showed statistically higher results than that of the young age group (64.2 ± 30.4 vs. 21.7 ± 9.8, *p* < 0.05), and facial TPD was higher in the old age group, but it was not statistically significant (12.4 ± 3.6 vs. 9.9 ± 3.5, *p* > 0.05). According to the TPD results, the values for dividing the forearm and facial participants into seven (sensitive group) and eight participants (low-sensitivity group) according to their sensitivities were 25.0 and 11.8, respectively.

#### 3.2.2. Numerical Rating Scale for Pain Caused by Stimulation

**Figure 6:**
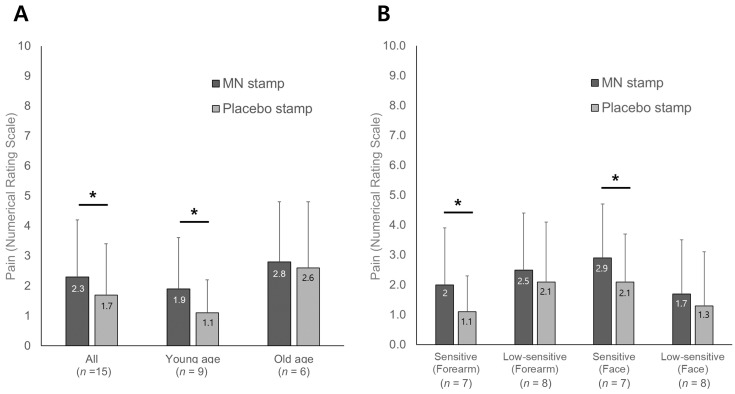
Numerical rating scale of pain. (**A**) Comparison according to age difference; (**B**) comparison according to sensory sensitivity; *, significant difference in MN and placebo stamp (*p* < 0.05); MN, microneedle.

Figure 6 and Supplementary Table S3 show the pain results immediately after stimulation, as evaluated subjectively by the participant. The average pain score for the MN stamp was 2.3 ± 1.9, and for the placebo stamp, it was 1.7 ± 1.7. Overall, the pain intensity was perceived as weak, and the MN stamp group had significantly higher pain than the placebo stamp group. As a result of the NRS comparison of the age difference, both the young and old age groups recognized that the MN stamps were more painful than the placebo stamps, with statistically significant differences only found in the younger age group (*p* = 0.013). A sub-analysis based on skin sensitivity showed that there was a significant difference in pain between the MN and placebo stamps only in the sensitive group for the forearm and face (*p* = 0.033 and *p* = 0.046, each).

#### 3.2.3. Blinding Indices

As a result of Bang’s BI, the BI of the MN stamp for all the participants was 0.00 (95% CI: −0.33, 0.33), which means that it was guessed randomly, but in the case of the placebo stamp, the BI was 0.30 (95% CI: −0.01 to 0.61), indicating that blinding was not successfully implemented. In subgroup analysis based on age, these trends were consistent (Table 2 and Table 3). In the case of sub-analysis based on skin sensitivity, successful blinding results, based on random guessing, were shown only for low-sensitivity groups on the forearm or face. Participants with sensitive forearm and facial sensations tended to guess the placebo stamp more accurately than those with low sensitivity (Table 4).

### 3.3. Needle Drawing of the Skin in Contact with the Stimulation Device

The shape of the bottom surface of the MN stamps or placebo stamps imagined by the participants was diverse based on the sensations they felt. The pictures drawn by the participants were categorized and presented in Supplementary Figure S3: (1) participants not only felt the difference in stimulation according to each part but also distinguished between the needle thickness of the MN or placebo stamps (Supplementary Figure S3A); (2) in the case of not being able to distinguish between stimulation devices but distinguishing stimulation areas (Supplementary Figure S3B); (3) felt the same or expressed the same for all stimulations (Supplementary Figure S3C); or (4) expressed differently according to the stimulation of each area.

## 4. Discussion

This study introduced a placebo control for the MN stamp and assessed its validity for blinding. The treatment intervention involved a 0.5 mm MN stamp with 42 needles, while the control was a placebo stamp with four non-penetrating acupressure-type needles. Using 3D printing, identical appearances were created for both stamps. Initial tests ensured that the placebo stamp did not penetrate the skin, and a suitable pressure (0.6 kgf) for skin irritation was established. A blinding test was conducted on 15 participants across four areas on the forearm and face, randomly applying MN or placebo stamps. While both interventions resulted in low reported pain levels, the MN stamp induced slightly more pain. The overall analysis using Bang’s BI revealed that the MN stamp was successfully blinded (random guess), whereas the placebo stamp was unblinded. However, subgroup analysis based on skin sensitivity demonstrated successful blinding for both interventions in the low-sensitivity group. There were no significant differences in blindness according to age. This study is meaningful in proposing a placebo control for MN stamps, which has not yet been reported, and in evaluating its validity. In particular, this study proposed a placebo model targeting the physical stimulation of MN in contrast to the placebo control groups of MNs, which mainly used drugs as placebos [31].

Our placebo control for the MN stamps was devised to use the low sensitivity of the skin to cause similar sensations without penetrating the skin. This method has also been proposed in previous acupuncture control studies [32]. Existing impenetrable sham acupuncture has several limitations, including potential sensations due to ‘deqi’ [33], incomplete inactivity [34], and potential bias in participants familiar with acupuncture [35]. The MN stamp used in this study had a larger number of needles and weaker stimulation than existing acupuncture. Therefore, even if the physical stimulation of the placebo stamp was weak, the participants were expected to be sufficiently blinded. However, when a participant was stimulated with both interventions at different sites (forearm or face), it could be confirmed that the blinding was broken in the part that distinguished whether it was an MN or placebo stamp, although the exact number of needles and shape were not clearly recognized (Supplementary Figure S3). Subgroup analysis revealed that participants less sensitive to the TPD were blinded by the placebo stamp, suggesting a correlation between skin sensitivity and successful blinding. In general, it is not possible to deliberately select participants with low-sensitivity skin for an MN-randomized placebo-controlled trial. To overcome this challenge, as in previous placebo acupuncture control studies [18], it may be necessary in MN-controlled studies to attempt to analyze the placebo effect of treatment intervention by investigating the indicators in advance, such as the participant’s existing experience with treatment or anticipation of treatment. However, we still need to recognize that the changes that acupuncture and MNs cause in patients may be distinctly different, and future studies are needed to confirm this. In addition, when using the same type of control group as in this study, it should be considered that skin sensitivity can also be an important variable. In future blinded test studies, TPD analysis should be included in the process of recruiting participants, and it is necessary to compare and analyze the TPD results according to the blinding results.

This study provides several implications for consideration when proposing placebo controls for the MN stamps. First, there are many different types of MNs, with variations in needle thickness, number, and skin contact area, and different regulations for medical and cosmetic use, making it difficult to choose a standard MN stamp to utilize for control development. In terms of needle length, MNs of various lengths are being utilized, ranging from 25–50 μm to 1500–2500 μm [36,37,38,39]. To address this, we conducted a comprehensive review of clinical studies and commercial MN stamps [40,41], setting the experimental group with a 500 μm length, commonly found in medical collagen-inducing therapy device approved by the Food and Drug Administration (FDA) [42]. However, for cosmetic models with lengths predominantly 200 μm or less [6], further blinding assessments are required. Determining an appropriate placebo control group depends on aligning with the standard MN stamp relevant to the research objectives. Second, it is necessary to consider various methods for evaluating skin penetration. Diverse methods exist for assessing skin penetration following MN insertion, including transepidermal water loss, electrical resistance [43], and post-penetration staining [44]. Previous studies employed trypan blue [45], gentian violet [46], and MBD [24] for post-penetration staining. Methylene blue dye is mainly used for skin sample studies, whereas gentian violet is favored for human penetration studies [47]. Given the concerns about skin necrosis in breast cancer patients using MBD through intradermal injection [22], we excluded participants with breast cancer-related conditions. Future research should establish a protocol for selecting appropriate evaluation methods for skin invasion with MN stamps. Third, careful consideration is needed in selecting the MN stamp stimulation area. In this study, the forearm and nasolabial fold on the face were chosen based on previous vaccine delivery [48] and facial wrinkle studies [14]. However, stimulation locations can vary widely in cosmetic and drug delivery studies, depending on the study’s purpose and the targeted disease. For instance, facial wrinkle studies often include the periorbital fold in addition to the nasolabial fold [49,50], while skin brightening studies focus on the T- and U-zone [51] and cheek areas [52]. Alopecia studies commonly stimulate the scalp area [53,54], and drug delivery research may prioritize the abdomen [55], deltoid [8,56], and thigh [57]. Future blind test studies with placebo control groups should tailor the stimulation area to each study’s specific purpose and perform a thorough comparison. Fourth, when proposing a placebo model for MN stamps that focused on physical stimulation, skin invasion was not the only answer. Before designing the placebo model used in this study, we considered inducing sensations similar to MNs through low-temperature stimulation. However, technological limitations in manufacturing a placebo model based on temperature led to its exclusion due to challenges in maintaining an immediate low temperature and potential performance bias in the contact area. A placebo study through heat transfer has been reported [58], but placebo technology through low temperature has not been reported. Future research should explore technologies capable of delivering instant low temperatures that induce a pain-like feeling without causing physiological reactions. Overcoming these technical limitations could extend the applicability of such models, not only for MNs but also for acupuncture research.

This study has several limitations. Firstly, technical constraints in the 3D printing process led to variations in the number and spacing of needles in the placebo MN stamp in the process of defining the thickness and spacing of the needles (Figure 2). Secondly, the small participant sample size hindered interpreting the relationship between the stimulation site, strength, and invasion rate. Lastly, physiological responses beyond pain and BI were not explored. While the noninvasive nature of the placebo stamp prevents physiological reactions related to microchannel formation and transdermal collagen induction caused by MN stamp skin invasion, additional research is required to analyze differences in physiological responses between the two devices [59].

## 5. Conclusions

This is the first study to propose a non-invasive placebo control that could be used to evaluate the efficacy of the MN stamp. We configurated a placebo control that is similar in appearance to the MN stamp but has fewer needles and a blunt, thicker tip that does not penetrate the skin. We experimentally confirmed that the placebo MN stamp does not invade the skin in healthy participants. Based on our assessment of the blinding of the participants, the success of the placebo control for the MN stamp proposed in this study was particularly limited to participants with low skin sensitivity. In future clinical placebo control trials, baseline information on the skin sensitivity of the participants should be collected and considered in the analysis, and a variety of appropriate placebo controls for different MN therapeutic interventions should be explored.

## Figures and Tables

**Table 1:** Characteristics of patients included in study.

	All Age (*n* = 15)	Young Age Group (*n* = 9)	Old Age Group (*n* = 6)	Differences by Age Group (*p*-Value)
Age [mean ± SD, (range)]	42.3 ± 20.2 (23–71)	26.6 ± 1.6 (23–28)	65.8 ± 4.8 (60–71)	0.027 *
Male [*n* (%)]	6.0 (40)	5.0 (55.6)	1.0 (16.7)	0.046 *
Height (mean ± SD, cm)	165.2 ± 7.3	167.8 ± 6.8	161.3 ± 6.7	0.249
Weight (mean ± SD, kg)	63.4 ± 14.6	65.8 ± 17.5	59.8 ± 9.1	0.600
BMI (mean ± SD, kg/m^2^)	23.1 ± 4.2	23.2 ± 5.3	22.9 ± 2.2	0.917
Drinking [%, (*n*^a^/wk)]	0.5 (0.6 ± 0.8)	0.7 (1.0 ± 0.9)	0.1 (0.2 ± 0.4)	0.157
Smoking [%, (*n*^b^/day)]	0.1 (10)	0.1 (10)	0.1 (10)	1.000
TPD arm (mm, mean ± SD)	38.7 ± 29.2 #	21.7 ± 9.8 #	64.2 ± 30.4 #	0.003 *
TPD face (mm, mean ± SD)	10.9 ± 3.7	9.9 ± 3.5	12.4 ± 3.6	0.135
TPD total (mm, mean ± SD)	24.8 ± 25.0	15.8 ± 9.4	38.3 ± 33.9	0.013 *

*, significant (*p* < 0.05) difference in young age group and old age group; #, significant (*p* < 0.05) difference in TPD of arm and TPD of face; BMI, body mass index; *n*^a^, number of drinking times a week; *n*^b^, number of cigarettes per day; TPD, two-point discrimination; SD, standard deviation.

**Table 2:** Number of participants’ guesses for each type of stamp.

Stimulation Site	Types of Stimulated Stamp	Guessed MN Stamp	Guessed Placebo Stamp	Don’t Know	Total
Face site	MN	7 (23.3%) *	6 (20.0%)	2	15
	Placebo	4 (13.3%)	8 (26.7%) *	3	15
	Total	11	14	5	30
Forearm site	MN	6 (20.0%) *	7 (23.3%)	2	15
	Placebo	4 (13.3%)	9 (30.0%) *	2	15
	Total	10	16	4	30
All site	MN	13 (21.7%) *	13 (21.6%)	4	30
	Placebo	8 (13.3%)	17 (28.3%) *	5	30
	Total	21	30	9	60

*, the numbers that the participants correctly guessed; MN, microneedle.

**Table 3:** Blinding index and blinding status for each type of stamps.

Stimulation Site	Types of Stimulated Stamp	All Age (*n* = 15)		Young Age Group (*n* = 9)		Old Age Group (*n* = 6)	
		BI (95% CI)	Blinding Status	BI (95% CI)	Blinding Status	BI (95% CI)	Blinding Status
Face site	MN	0.07 (−0.4 to 0.54)	Random guess	−0.13 (−0.77 to 0.52)	Random guess	0.50 (−0.11 to 1.11)	Unblinded
	Placebo	0.27 (−0.17 to 0.7)	Unblinded	0.38 (−0.22 to 0.97)	Unblinded	0.00 (−0.65 to 0.65)	Unblinded
Forearm site	MN	−0.07 (−0.54% to 0.4)	Random guess	−0.30 (−0.86 to 0.26)	Opposite guesses	0.29 (−0.37 to 0.94)	Unblinded
	Placebo	0.33 (−0.11 to 0.77)	Unblinded	0.30 (−0.26 to 0.86)	Unblinded	0.60 (−0.10 to 1.30)	Unblinded
All site	MN	0.00 (−0.33 to 0.33)	Random guess	−0.20 (−0.65 to 0.20)	Random guess	0.33 (−0.15 to 0.81)	Unblinded
	Placebo	0.30 (−0.01 to 0.61)	Unblinded	0.33 (−0.07 to 0.74)	Unblinded	0.25 (−0.22 to 0.72)	Unblinded

BI, blinding index; CI, confidence intervals; MN, microneedle.

**Table 4:** Blinding index and blinding status for sensory of forearm and face sites.

Groups	Stimulation Site	Types of Stimulated Stamp	Low-Sensitive Group (*n* = 8)		Sensitive Group (*n* = 7)	
			BI (95% CI)	Blinding Status	BI (95% CI)	Blinding Status
Based on sensory of forearm	Forearm site	MN	0.00 (−0.69 to 0.69)	Random guess	−0.13 (−0.77 to 0.52)	Random guess
		Placebo	0.00 (−0.69 to 0.69)	Random guess	0.63 (0.14 to 1.11)	Unblinded
	All site	MN	−0.14 (−0.62 to 0.34)	Random guess	0.13 (−0.33 to 0.58)	Random guess
		Placebo	0.14 (−0.34 to 0.62)	Random guess	0.44 (0.05 to 0.82)	Unblinded
Based on sensory of face	Face site	MN	0.13 (−0.52 to 0.77)	Random guess	0.00 (−0.69 to 0.69)	Random guess
		Placebo	−0.25 (−0.82 to 0.32)	Opposite guesses	0.86 (0.60 to 1.12)	Unblinded
	All site	MN	0.00 (−0.46 to 0.46)	Random guess	0.00 (−0.48 to 0.48)	Random guess
		Placebo	−0.06 (0.50 to 0.38)	Random guess	0.71 (0.41 to 1.02)	Unblinded

BI, blinding index; CI, confidence intervals; MN, microneedle.

## Data Availability

Data are contained within the article.

## Supplementary Materials

The following supporting information can be downloaded at: https://www.mdpi.com/article/10.3390/pharmaceutics16030395/s1, Figure S1: Five stimulation positions of the forearm to confirm microneedles skin penetration; Figure S2: Number of stained spots caused by needle penetration according to stimulation intensity (force of pressing) in the microneedle stamp group. The bar graph represents the number of stained spots caused by needle penetration and line graph depicts the stimulation intensity. Figure S3: MNs stamp and placebo stamp picture representation based on skin contact. (A) Participant-drawn drawings showing the differences in sensation of needle thickness for MNs stamps and placebo stamps (Subjects No. 4 and 6); (B) Participant-drawn drawings showing the differences in sensation of stimulation area (Subject No. 3); (C) Participant-drawn drawings showing uniform stimulation sensation for all stimulation sites and for all types of stamps (Subject, No. 12); MNs, microneedles. Table S1: Differences in main parameters for each type of stamps. Table S2: Correlational matrix between stimulation site, stimulation intensity (force), and number of invaded points. Table S3: Numerical rating scale of pain.
