# Supplementary material for: Designing a Placebo Microneedle Stamp: Modeling and Validation in a Clinical Control Trial
Source: Pharmaceutics. 2024 Mar 14;16(3):395. doi: 10.3390/pharmaceutics16030395 (PMC10975904; doi:10.3390/pharmaceutics16030395)
Supplement: Supplementary file 1 [file pharmaceutics-16-00395-s001.zip › pharmaceutics-2888072-supplementary.pdf]

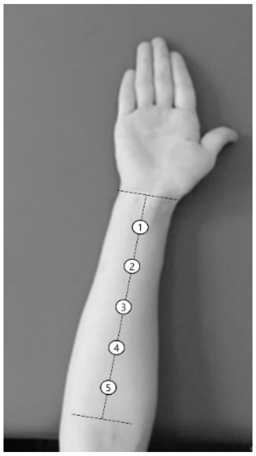

Figure S1. Five stimulation positions of the forearm to confirm microneedles skin penetration.

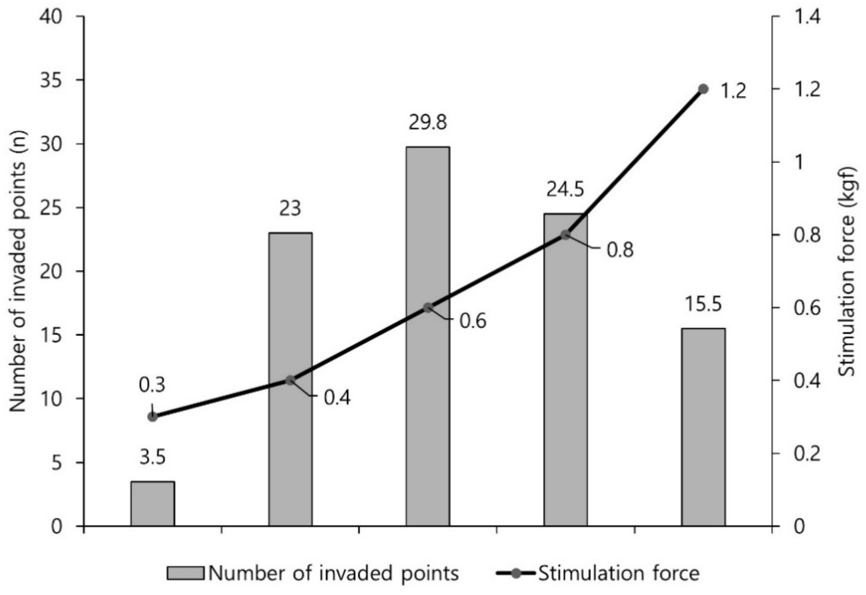

Figure S2. Number of stained spots caused by needle penetration according to stimulation intensity (force of pressing) in the microneedle stamp group. The bar graph represents the number of stained spots caused by needle penetration and line graph depicts the stimulation intensity.

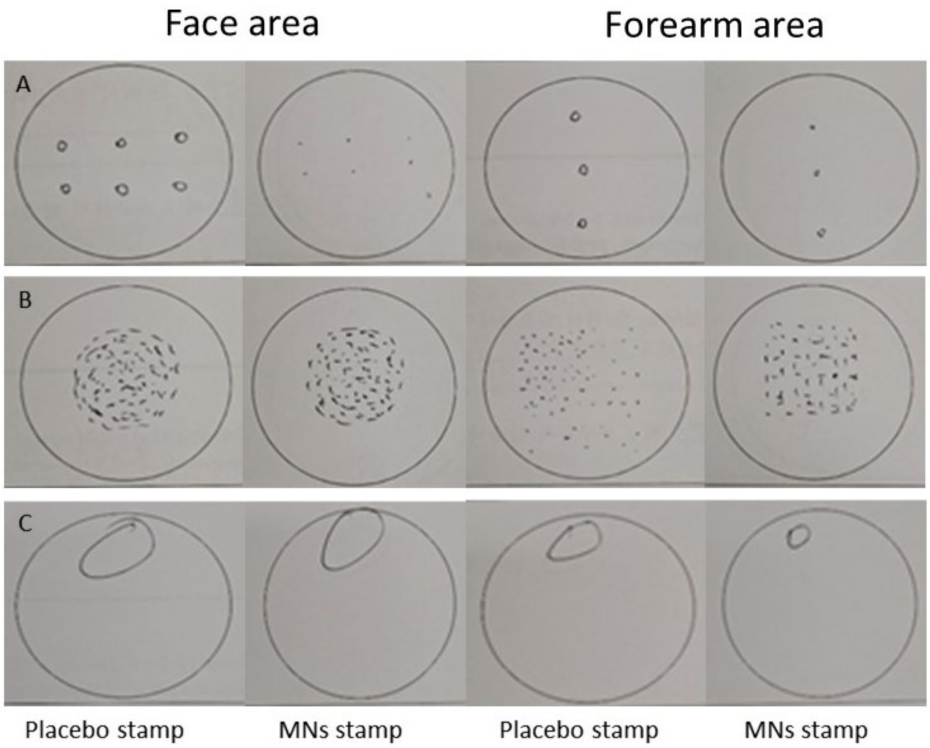

Figure S3. MNs stamp and placebo stamp picture representation based on skin contact. (A) Participant-drawn drawings showing the differences in sensation of needle thickness for MNs stamps and placebo stamps (Subjects No. 4 and 6); (B) Participant-drawn drawings showing the differences in sensation of stimulation area (Subject No. 3); (C) Participant-drawn drawings showing uniform stimulation sensation for all stimulation sites and for all types of stamps (Subject, No. 12); MNs, microneedles.

Table S1. Differences in main parameters for each type of stamps.

	MN Stamp	Placebo MN Stamp
Appearance of needle	Identical appearances (using 3D printing technology)	
Shape of needle	Triangular pyramid-shape	
Skin contact area	A circle with a diameter of 14 mm	
Penetration	Penetration	Non-penetration
Number of needles	42 needles	4 needles
Length of needle	0.5 mm	1 mm
Diameter of needle	< 0.7 mm	0.7 mm
Interval of needle	< 0.5 mm	0.5 mm

MN, microneedle.

Table S2. Correlational matrix between stimulation site, stimulation intensity (force), and number of invaded points.

Variables	Site	Stimulation force	Number of invaded points
Site	1.000	0.295	-0.302
Stimulation force	0.295	1.000	-0.588
Number of invaded points	-0.302	-0.588	1.000

*, p<0.05; all variables p>0.05

Table S3. Numerical rating scale of pain.

		MN stamp (mean ± SD)	Placebo stamp (mean ± SD)	P-value
All participants (*n* = 15)		2.3 ± 1.9	1.7 ± 1.7	0.008*
Subgroup (age)	Young age group (*n* = 9)	1.9 ± 1.7	1.1 ± 1.1	0.013*
	Old age group (*n* = 6)	2.8 ± 2.0	2.6 ± 2.2	0.317
Subgroup (sensory sensitivity)	Low-sensitive group (Forearm, *n* = 8)	2.5 ± 1.9	2.1 ± 2.0	0.107
	Sensitive group (Forearm, *n* = 7)	2.0 ± 1.9	1.1 ± 1.2	0.033*
	Low-sensitive group (Face, *n* = 8)	1.7 ± 1.8	1.3 ± 1.8	0.084
	Sensitive group (Face, *n* = 7)	2.9 ± 1.8	2.1 ± 1.6	0.046*

*, significant ($p<0.05$) difference in MN stamp and placebo stamp; MN, microneedle; SD, standard deviation.
